# Snake Venomics and Antivenomics of Cape Cobra (*Naja nivea*) from South Africa: Insights into Venom Toxicity and Cross-Neutralization Activity

**DOI:** 10.3390/toxins14120860

**Published:** 2022-12-07

**Authors:** Choo Hock Tan, Kin Ying Wong, Li-Kun Huang, Kae Yi Tan, Nget Hong Tan, Wen-Guey Wu

**Affiliations:** 1 Venom Research and Toxicology Laboratory, Department of Pharmacology, Faculty of Medicine, University of Malaya, Kuala Lumpur 50603, Malaysia; 2 Institute of Bioinformatics and Structural Biology, Department of Life Science, National Tsing Hua University, Hsinchu 300044, Taiwan; 3 Protein and Interactomics Laboratory, Department of Molecular Medicine, Faculty of Medicine, University of Malaya, Kuala Lumpur 50603, Malaysia

**Keywords:** proteomics, neurotoxic envenoming, immunoreactivity, immunorecognition, toxins, polyvalent antivenom

## Abstract

*Naja nivea* (Cape Cobra) is endemic to southern Africa. Envenoming by *N. nivea* is neurotoxic, resulting in fatal paralysis. Its venom composition, however, has not been studied in depth, and specific antivenoms against it remain limited in supply. Applying a protein decomplexation approach, this study unveiled the venom proteome of *N. nivea* from South Africa. The major components in the venom are cytotoxins/cardiotoxins (~75.6% of total venom proteins) and alpha-neurotoxins (~7.4%), which belong to the three-finger toxin family. Intriguingly, phospholipase A_2_ (PLA_2_) was undetected—this is a unique venom phenotype increasingly recognized in the African cobras of the *Uraeus* subgenus. The work further showed that VINS African Polyvalent Antivenom (VAPAV) exhibited cross-reactivity toward the venom and immunorecognized its toxin fractions. In mice, VAPAV was moderately efficacious in cross-neutralizing the venom lethality with a potency of 0.51 mg/mL (amount of venom completely neutralized per milliliter of antivenom). In the challenge-rescue model, VAPAV prevented death in 75% of experimentally envenomed mice, with slow recovery from neurotoxicity up to 24 h. The finding suggests the potential para-specific utility of VAPAV for *N. nivea* envenoming, although a higher dose or repeated administration of the antivenom may be required to fully reverse the neurotoxic effect of the venom.

## 1. Introduction

Snakebite envenoming is a priority neglected tropical disease designated by the World Health Organization [1]. Each year, it causes 81,000–138,000 deaths and three times as many chronic complications worldwide [2]. Sub-Saharan Africa is one of the most affected regions, with nearly 500,000 cases resulting in more than 30,000 deaths annually. Worse still, this is accompanied by a countless toll of permanent disabilities and amputations [3,4]. The annual burden of snakebite has been recently estimated at 1.03 million DALYs (disability-adjusted life years), an astonishing figure that is close to or even higher than the burden of many Neglected Tropical Diseases (NTDs) [5].

In Africa, there are approximately 400 snake species, of which 135 are medically important and capable of causing life-threatening envenoming [6]. Cobras (*Naja* spp.) are implicated in most bites and responsible for high fatalities. The African cobras are represented by members of three subgenera: *Afronaja* (African spitting cobras), *Boulengerina* (water cobras) and *Uraeus* (African non-spitting cobras) [7]. Of these, Cape cobra (*Naja nivea*), along with species within the *Naja haje* (Egyptian cobra) complex (now constituting *N. haje*, *Naja senegalensis*, *Naja arabica*, *Naja annulifera* and *Naja anchietae*), are grouped under the subgenus of *Uraeus* [7]. Cape cobra diverged from the *Naja haje* complex earlier, presumably following allopatric speciation, and has since remained endemic to the southern part of the African continent. On the other hand, the *N. haje* complex species are more widely distributed in the north of the continent. *N. nivea* is considered a deadly snake of high medical importance (WHO Category 1) due to its common occurrence and its bite being associated with high mortality in South Africa, South Botswana, and Namibia [8,9].

Earlier studies from the 1950s to the 1980s showed that *N. nivea* venom contained neurotoxins and cardiotoxins, and exhibited neurotoxic and cardiotoxic activities in animals [10,11,12,13]. However, a comprehensive venom analysis, including proteomics of *N. nivea* venom, remains unavailable, hindering a deeper understanding of the pathophysiology of envenoming by this species. Clinically, it has been reported that envenoming by *N. nivea* led to severe neurotoxicity, where complete flaccid paralysis occurred within 3 h following a bite, and the victim required mechanical ventilation for a prolonged duration of 4–7 days [14]. The cases were treated with an African antivenom (SAIMR Polyvalent Antivenom) which was raised against 10 species of viperid and elapid snakes, including *N. nivea*). It was reported that the antivenom was unable to fully reverse the established neurotoxicity once the paralysis set in, suggesting low effectiveness of antivenom treatment in this case. More recent research has also concluded that various antivenom products marketed in sub-Saharan Africa indeed lack efficacy or that evidence is scarce to support their clinical use [15]. The South African Institute for Medical Research (SAIMR) Polyvalent Antivenom is commonly used to treat envenoming caused by *N. nivea* in South Africa, but its neutralization efficacy has just been shown to be low in another recent preclinical study [16]. The situation is further exacerbated by a shortage of antivenom supply in Africa, ostensibly due to resource scarcity and low profitability of the antivenom industry. To meet the market need, foreign manufacturers, especially those based in India, have been producing antivenoms for use in Africa, with products such as VINS African Polyvalent Antivenom (VAPAV). VAPAV is raised against 10 common African venomous snake species, i.e., *Naja melanoleuca*, *Naja nigricollis*, *N. haje*, *Dendroaspis polylepis*, *Dendroaspis viridis*, *Dendroaspis jamesoni*, *Bitis gabonica*, *Bitis arietans*, *Echis leucogaster*, and *Echis ocellatus*. Of these, *N. haje* is phylogenetically closely related to *N. nivea*, and therefore VAPAV is hypothetically able to cross-neutralize the venom of *N. nivea*. The para-specific utility of VAPAV, however, has not been investigated and verified. Hence, this study first set to unravel the composition of *N. nivea* venom through a decomplexation proteomic approach. Subsequently, the study examined the immunoreactivity of VAPAV toward the venom and its toxin fractions, and scrutinized the *in vivo* efficacy of the antivenom in cross-neutralizing the venom’s lethality.

## 2. Results

### 2.1. Decomplexation of N. nivea Venom by Reverse-Phase HPLC

**Figure 1:**
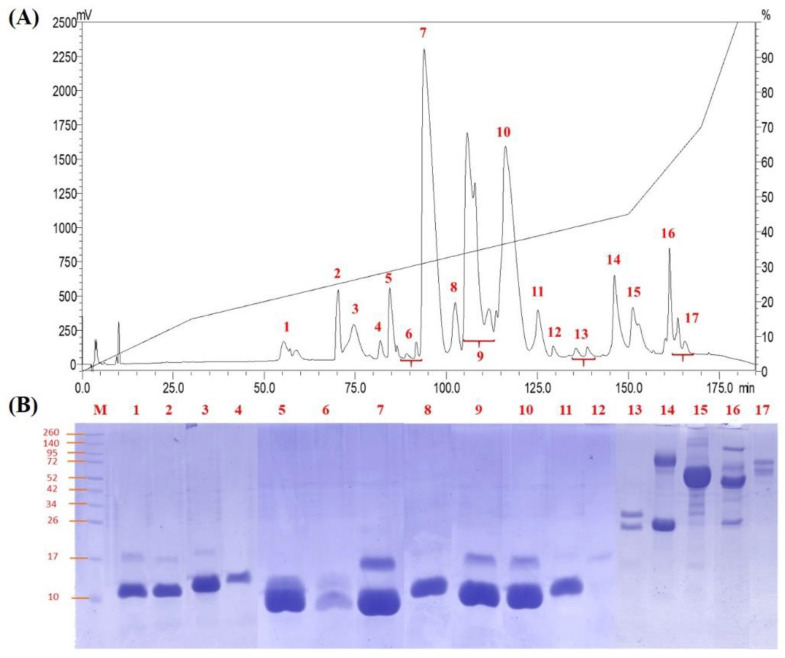
Chromatographic and electrophoretic profiles of *Naja nivea* venom. (**A**) Venom fractionation using C18 reverse-phase HPLC. (**B**) Gel electrophoresis of reverse-phase HPLC collected protein fractions (1–17) with 15% SDS-PAGE under reducing conditions. M indicates molecular markers.

The *N. nivea* venom was resolved by C18 reverse-phase HPLC, yielding 17 protein fractions, as shown in Figure 1A. Electrophoretic profiles showed fractions 1–12 (eluted between 55–130 min of HPLC) contained mainly low molecular weight proteins (7–16 kDa), which accounted for nearly 87% of total venom proteins (Figure 1B). Fractions 13–17 were minor proteins eluted in the later course of fractionation (beyond 130 min of HPLC), consisting of moderate to high molecular weight proteins (>20 kDa) in the venom.

### 2.2. Naja nivea Venom Proteome

**Figure 2:**
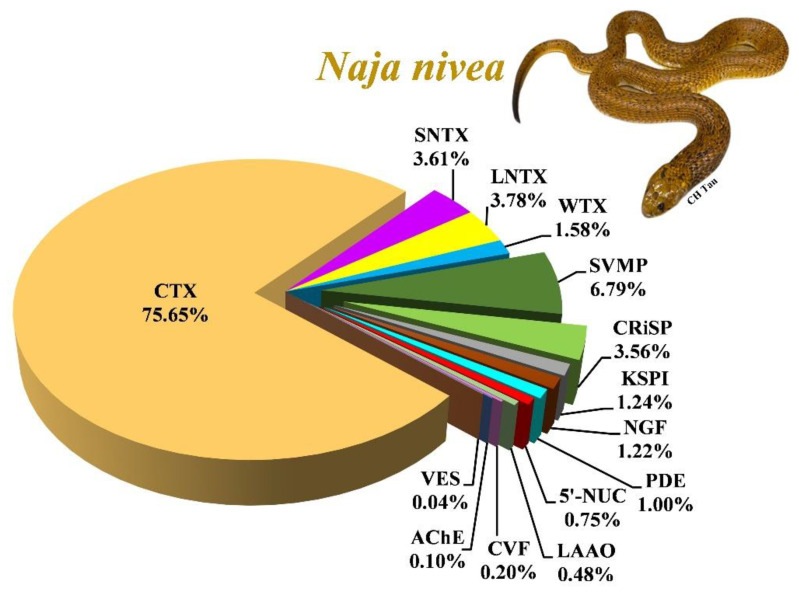
Proteome of South African *N. nivea* venom profiled using C18 RP-HPLC followed by liquid chromatography-tandem mass spectrometry. The 3FTx protein family consists of CTX, SNTX, LNTX and WTX. CTX is the major component (75.64% of the total venom proteins), followed by alpha-neurotoxins (both SNTX and LNTX, 7.43%). Abbreviations: 3FTx, three-finger toxins; SNTX, short neurotoxin; LNTX, long neurotoxin; WTX, weak neurotoxin; CRiSP, cysteine-rich secretory protein; SVMP, snake venom metalloproteinase; KSPI, Kunitz-type serine protease inhibitor; LAAO, L-amino acid oxidase; PDE, phosphodiesterase; AChE, acetylcholinesterase; CVF, cobra venom factor; NGF, nerve growth factor; 5′-NUC, 5′-nucleotidase; VES, vespryn; PLA_2_, phospholipase A_2_. Inset: African Cape Cobra, *N. nivea*.

The tandem mass spectrometry analysis identified a total of 58 proteoforms from all fractions of *N. nivea* venom (Table 1). Of these, 43 proteoforms were annotated based on peptide sequences that belonged to cobras (*Naja* spp.; Table 2; Supplementary File S1). These proteins were sorted and categorized into 11 protein families, along with their relative protein abundances (Figure 2). Three-finger toxins (3FTx) constitute the main protein family, accounting for 84.62% of total venom proteins in which cytotoxins/cardiotoxins (CTX, 75.65%) and α-neurotoxins (short and long neurotoxins, 7.39%) are abundantly expressed. Other proteins with lower abundances (collectively < 20% of total venom proteins) belong to the toxin families of snake venom metalloproteinase (SVMP), cysteine-rich secretory protein (CRiSP), Kunitz-type serine protease inhibitor (KSPI), nerve growth factor (NGF), phosphodiesterase (PDE), 5′-nucleotidase (5′-NUC), L-amino acid oxidase (LAAO), cobra venom factor (CVF), acetylcholinesterase (AChE) and vespryn (VES; Table 2). Mass spectrometric data, including parameters for spectral ions, protein scores and the amino acid sequences of peptides, are provided in the Supplementary Materials (Supplementary File S1).

### 2.3. Immunological Binding Activity of VAPAV toward N. nivea Venom

**Figure 3:**
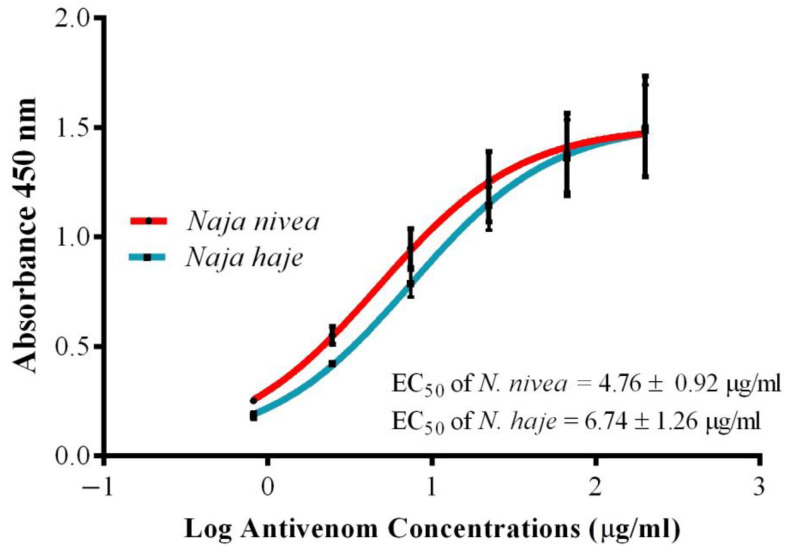
Immunoreactivity of VINS African Polyvalent Antivenom (VAPAV) toward the venoms of *N. nivea* and *N. haje*. *N. haje* venom was used as a positive control.

In indirect ELISA, the antivenom VAPAV exhibited concentration-dependent immunoreactivity toward the venoms of *N. nivea* and *N. haje* (positive control; Figure 3). The binding activities of VAPAV toward *N. nivea* and *N. haje* venoms were comparable, as indicated by the values of its maximal absorbance and half-maximal effective concentration (EC_50_) for the two venoms. Its EC_50_ for *N. nivea* venom was 4.76 ± 0.92 μg/mL, and for *N. haje* venom, 6.74 ± 1.26 μg/mL (*p* > 0.05).

### 2.4. Immunorecognition of N. nivea Venom Fractions by VAPAV

**Figure 4:**
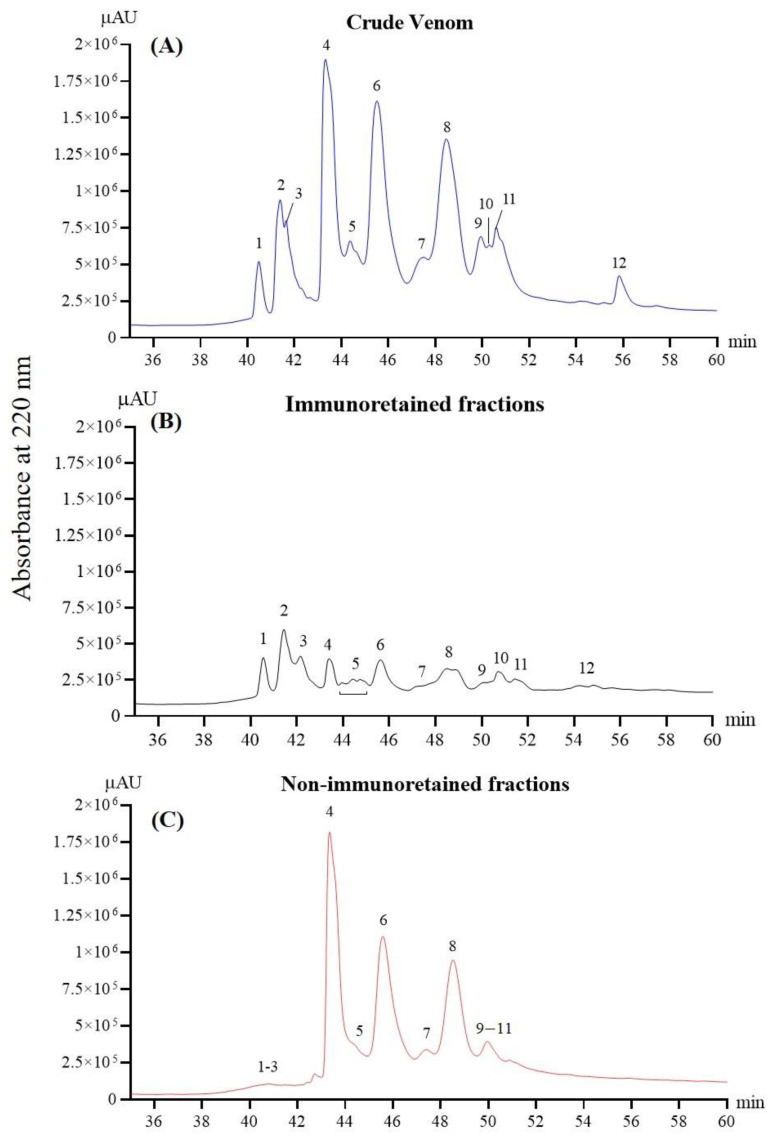
Immunorecognition of *N. nivea* venom proteins by VAPAV. Panels illustrate the reverse-phase HPLC separation of the following into fractions: (**A**) whole venom proteins; (**B**) venom proteins immunoretained by the VAPAV-immobilized affinity column; (**C**) venom proteins that were not bound by the immunoaffinity column.

The RP-HPLC profiles of the whole venom, immunoretained and non-immunoretained venom proteins, are shown in Figure 4 (panels A, B, and C, respectively). The result illustrates the antivenomics for VAPAV against *N. nivea* venom, where the antivenom was shown to immunorecognize and bind to the 12 protein fractions of venom at different degrees of immunoreactivity. Comparing between the profiles (Figure 4A–C), proteins in RP-HPLC fractions 1, 2, and 3, as well as the tailing fraction 12, were most markedly immunocaptured by VAPAV, whereas proteins in fractions 4–8 were least immunoretained. The degrees of immunoretention of venom proteins in these fractions were tabulated as percentages in Table 3. Accordingly, the VAPAV affinity column efficiently immunorecognized venom proteins in fractions 1, 2, and 3 (68–70% immunoretention). The binding activity of VAPAV for venom proteins in fraction 12 was moderate (~58% immunoretention) but low for those in fractions 4–11 (~20–25% immunoretention). The result of immunoretention was consistent with the corresponding non-immunoretained fractions, as shown in Figure 4C, where unbound proteins were washed out from the affinity column.

### 2.5. Venom Lethality and Neutralization by Antivenom

The intravenous median lethal dose (*i.v.* LD_50_) of *N. nivea* venom was determined to be 1.11 μg/g in mice (Table 4). Considering that VAPAV exhibited high immunoreactivity toward the whole venom and was able to immunorecognize most of its protein fractions, the *in vivo* neutralization efficacy of the antivenom was subsequently examined in mice. In the venom-antivenom preincubation test, VAPAV was able to cross-neutralize the lethality of *N. nivea* venom with a neutralization potency (P) of 0.51 mg venom per ml antivenom, equivalent to a normalized potency (n-P) of 3.90 mg venom per gram of antivenom protein (Table 4). For comparison, Table 4 also included EC_50_ values for the neutralization of *N. nivea* venom by other polyvalent antivenoms, i.e., products from the South African Institute for Medical Research (SAIMR), the Bioclon Institute of Mexico (Antivipmyn Africa), and the Egyptian Organization for Biological Products and Vaccines (VACSERA) based on previous reports. In the present work, the potency values of the different antivenom products were calculated based on their reported EC_50_, challenge doses and antivenom protein concentrations where applicable.

### 2.6. Experimental Envenoming and Rescue with Antivenom

**Figure 5:**
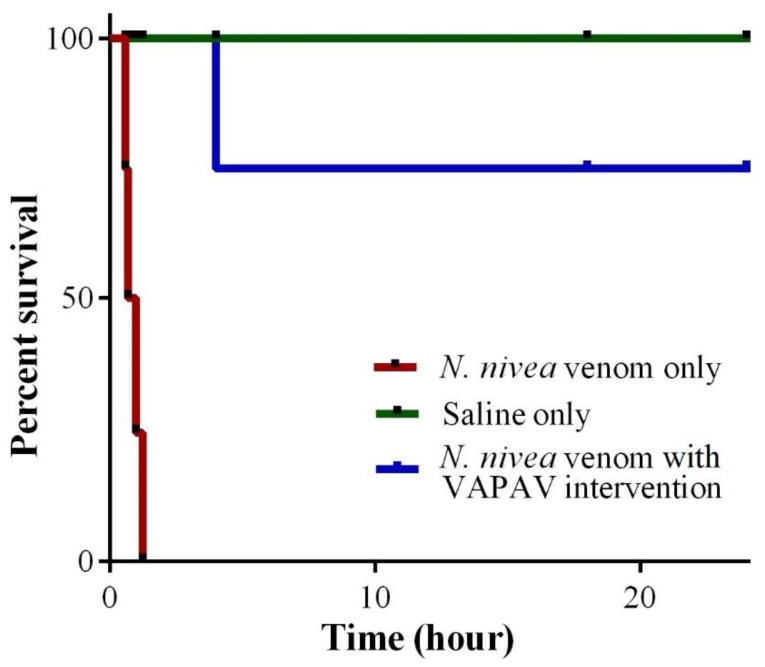
Survival plot of mice treated with VINS African Polyvalent Antivenom (VAPAV) in a challenge and rescue model of envenoming with *N. nivea* venom. Mice (*n* = 8 per group) were subcutaneously inoculated with 5 × LD_50_ of the venom. The rescue group received 250 µL VAPAV upon the onset of neurotoxicity at approximately 30 min.

The subcutaneous LD_50_ of *N. nivea* venom was 2.42 μg/g (95% C.I. 2.10–2.79) in mice, approximately one-fold higher than the intravenous LD_50_ (Table 4). In the experimental envenoming model, mice injected subcutaneously with 5 × *s.c.* LD_50_ *N. nivea* venom began to show weakness in movement within the first 30 min of envenoming. As the neurotoxicity progressed, the mice showed hind limb paralysis, movement difficulty, and labored breathing. In the group of untreated mice (*n* = 8), complete paralysis and death ensued within 2 h from the time of experimental envenoming (Figure 5). In the treated group, an intravenous bolus of VAPAV at 250 μL administered upon the onset of hind limb paralysis (approximately 30 min post-envenoming) rescued most of the mice (75% survival) from the lethality of *N. nivea* venom (Figure 5). Mice that were dead (25%) despite antivenom treatment had a marginally delayed death (dying by approximately 3–4 h post-envenoming) in comparison to the untreated mice, which succumbed to death between 1–2 h post-envenoming. The treated mice which survived the experimental envenoming (75%) showed a gradual reversal of paralysis with full recovery (regaining the ability to move and eat freely) observed by 24 h post-envenoming.

## 3. Discussion

In Africa, *N. nivea* bites are a cause of neurotoxic envenoming [9,14]. Applying a protein decomplexation approach, the present study unveiled the first venom proteome of *N. nivea*, showing the venom composition is predominantly three-finger toxins comprised of α-neurotoxins (α-NTX), cytotoxins/cardiotoxins (CTX) and weak neurotoxins (WTX). The α-NTX, though constituting only ~7.4% of the total venom protein, are the principal toxins responsible for neurotoxicity in *N. nivea* envenoming [14]. The α-NTX of *N. nivea* has also been shown to be highly lethal in mice with an *i.v* LD_50_ of 0.08 μg/g [20], similar to the α-NTX of various cobra species with *i.v* LD_50_ of approximately 0.1 μg/g in mice (for instance: [21,22,23]. The high lethality of *N. nivea* α-NTX is primarily a sequel of the post-synaptic blockade of nicotinic acetylcholine receptors (nAChR), which disrupts the neuromuscular transmission, resulting in paralysis and respiratory failure [24]. The lethal potency of cobra venom, nonetheless, is also modulated by the amount of these neurotoxins in the venom, as it has been established that the relative abundances of α-NTX in cobra venoms are strongly correlated with the venom’s lethality [25,26]. In principle, cobra venoms with a high α-NTX abundance (>20% of total venom proteins) have low LD_50_s below 0.2 μg/g (thus more lethal), while those containing less α-NTX (<10%) tend to have high LD_50_ above 1 μg/g (thus less lethal), in mice [27,28]. African cobras such as *N. nubiae*, *N. senegalensis*, and *N. melanoleuca* have venoms containing 12–20% α-NTX, and moderate lethal activity with an intermediate *i.v.* LD_50_ (≤0.6 μg/g) in mice [19,29,30]. On the other hand, *N. nivea* (present study), as with *N. nigricollis*, *N. katiensis*, *N. pallida*, *N. mossambica*, and *N. annulifera* have venoms containing α-NTX below 10% of total venom proteins, and therefore higher *i.v.* LD_50_s (0.9–2.4 μg/g) in mice [30,31]. Despite the somewhat low lethal potency of venom, *N. nivea* envenoming causes rapid and profound neurotoxicity clinically, which could be attributed to the large amount of venom injected in a bite (considering its medium-to-large body size of up to 1.8 m in length [32]), and the venom’s low human equivalent LD_50_ (0.09 mg/kg) upon allometric conversion. The other toxins with known neurological effects were named weak toxins (WTX), previously shown to have weak affinity toward neuronal and muscarinic receptors and low toxicity in mice (LD_50_, 5–80 mg/kg) [33,34]. The low abundance of WTX in *N. nivea* venom indicates its ancillary role in the pathophysiology of envenoming.

Cardiotoxins, interchangeably known as cobra cytotoxins (CTXs), are the most abundant toxins identified in the *N. nivea* venom proteome (75% of the total venom proteins). The high expression of CTX is consistent with proteomic findings in other African cobras from various subgenera, including *Afronaja* [30], *Boulengerina* [29], and *Uraeus* [19,31]. Compared with the α-NTX, cobra CTXs are relatively less potent in lethal activity, with higher *i.v.* LD_50_ beyond 1 μg/g in mice [22,23,35]. CTXs are usually implicated in local tissue necrosis and venom ophthalmia due to their cytolytic effect, which can be potentiated by synergistic PLA_2_ in snake venom [36,37]. The cytolytic activity of CTX varies between CTX proteoforms depending on the amino acid residue present at the tip of the protein’s loop II. Based on this characteristic, CTXs are categorized as P-type (Pro30) or S-type (Ser28). Studies on CTX interaction with model lipid membranes demonstrated that the P-type CTXs are more cytolytic than the S-type [38,39], while the latter (S-type CTX) has also been shown to be cytotoxic toward human cell lines [40]. The current study identified three CTX proteoforms, in agreement with the earlier report of three *N. nivea* cytotoxins (designated as V˝1, V˝2 and V˝3) whose amino acid sequences have been determined and deposited in the database as CTX 1 (Uniprot KB: P01456, S-type), CTX 2 (P01463, P-type) and CTX 3 (P01458, S-type), respectively [13]. Earlier studies, however, demonstrated that *N. nivea* venom and its CTXs (V˝1, V˝2, and V˝3) had rather weak hemolytic activity *in vitro* [13,41], and local necrosis has not been reported in *in vivo* experiments as well as in clinical envenoming cases [14,41]. The present study re-investigated the local tissue-damaging effect of *N. nivea* venom by inoculating it intradermally in C57BL/6 mice (*n* = 4 per dose), and found no dermonecrosis even at the highest sublethal dose tested (50 µg venom per mouse; data not shown). The finding suggests a lesser role of *N. nivea* CTX in the pathogenesis of local tissue necrosis or that the CTXs possibly exhibit other toxic activities, such as cardiotoxicity. Cardiotoxicity is considered a rare complication of cobra bite through disrupting the calcium regulation or mitochondria fragmentation in cardiac muscle cells, resulting in cardiac arrhythmia [42,43]. Earlier studies showed that *N. nivea* venom caused an unusual form of cardiac failure (systolic arrest of the ventricle) ex vivo in isolated perfused mammalian heart preparation [24,41], but this toxicity has not been well documented in human envenoming cases. It is possible that the abundant CTXs interact in synergism with other toxins present in the venom, thereby contributing to the overall toxicity of the venom. Further studies are warranted to elucidate the biological activity of *N. nivea* CTX and its correlation with the pathophysiology of envenoming.

In addition to 3FTx, other components detected in *N. nivea* venom were of low abundances (3–7%). These include snake venom metalloproteinases (SVMP) and cysteine-rich secretory proteins (CRiSP). Cobra SVMPs are zinc-dependent multidomain enzymes that may be instrumental in the activation of the complement system and inflammatory response to envenoming, contributing to the venom’s toxicity [44]. CRiSP is a common component in snake venoms with diverse biological activities such as inhibition of smooth muscle contraction, blockade of various cation channels, and induction of hypothermia in prey animals [45,46]. Other minor venom components (<2%) include Kunitz-type serine protease inhibitor (KSPI), nerve growth factor (NGF), phosphodiesterase (PDE), 5′-nucleotidase (5′-NUC), L-amino acid oxidase (LAAO), cobra venom factor (CVF), acetylcholinesterase (AChE), and vespryn (VES). KSPI are small proteins (5–7 kDa) that may be involved in diverse biological activities such as blood coagulation, fibrinolysis, inflammation, and blockade of ion channels [47,48]. NGF possibly acts as an inhibitor of metalloproteinase, preventing SVMP autodigestion [49]. Snake venom PDE and 5′-NUC can act on ATP and AMP molecules to release adenosine, thus facilitating venom spread in envenomed subjects [50,51]. CVF is also known to facilitate venom spread through the activation of the complement system, thus increasing inflammation and vascular permeability [52,53]. LAAO and AChE are both large enzymatic proteins in cobra venoms. Snake venom LAAO has recently been shown to stimulate the production of inflammatory mediators, contributing to local inflammatory effects in snakebite envenoming [54]. AChE may potentiate venom-induced neurotoxicity through the degradation of the neurotransmitter acetylcholine at the neuromuscular junction [55]. VES, first discovered in King Cobra venom, has been shown to induce hyperalgesia and hypolocomotion in prey animals [56]. Although these proteins are classified as putative venom toxins, their exact roles in specific cobra envenoming await further elucidation.

Intriguingly, the present study revealed a lack of phospholipase A_2_ (PLA_2_) in the venom proteome of *N. nivea*. Although PLA_2_ has long been regarded as a ubiquitous venom component across all snake lineages, proteomic and biochemical studies increasingly showed African non-spitting cobras from the *Uraeus* subgenus have venoms that contain little to no PLA_2_ [19,31,57,58]. This is a unique venom phenotype not shared by other African and Asiatic cobras whose venoms contain a significant amount of PLA_2_ in general (12–20% of total venom proteins) [25,29,30,59,60]. It is speculated that the loss of PLA_2_ in its venom evolution partly results in the lack of cytotoxic and necrotizing properties of *N. nivea* venom despite containing abundant CTX, since PLA_2_ and cobra CTX (known as direct lytic factor previously) have been known for a long time to act in synergism [36]. Further studies are needed to elucidate the cause and mechanism (probably through pseudogenization) as well as the evolutionary impact of PLA_2_ loss in the venom.

In sub-Saharan Africa, resource allocation and subsequent antivenom supply, access, affordability, and availability are inadequate [61]. Domestic antivenoms are high-priced and limited in supply. Antivenoms produced outside Africa (for instance, products procured from India targeting the African snake species) are increasingly imported into Africa since their supply is consistent and the price is low. There is, however, limited data to support the clinical effectiveness of their use in Africa [15]. In this study, we scrutinized the immunoreactivity and neutralization efficacy of VAPAV, a polyvalent antivenom developed in India for use in Africa, as a para-specific antivenom against *N. nivea* venom. The immunological binding activity of VAPAV toward *N. nivea* venom was comparable to the homologous *N. haje* venom (the venom used in hyperimmunizing the horses), implying conserved venom protein antigenicity between the two sister species which share close phylogenetic relatedness within the subgenus of *Uraeus*. On antivenomics, VAPAV was also found to have considerably good immunorecognition capacity for the venom protein fractions that were eluted in the initial course of RP-HPLC (fractions 1, 2 and 3, approximately 70% of protein binding capacity). These fractions represent alpha-neurotoxins as profiled by C18 revere-phase HPLC in the proteomic study, which is also consistent with the decomplexed venom profiles of other cobra species [21,25,29,30,60], including *N. annulifera* and *N. senegalensis* from within the *N. haje* complex itself [19,31]. The immunoretention of these neurotoxins indicated the cross-neutralization potential of VAPAV against *N. nivea* venom-induced neurotoxicity. As such, the *in vivo* neutralization activity of VAPAV against *N. nivea* venom was investigated, applying the venom and antivenom preincubation protocol for immunocomplexation as per the WHO guideline [62]. Consistent with the immunoreactivity finding, VAPAV was moderately effective in cross-neutralizing the venom’s lethal effect in mice. The parameter “normalized potency” was then used to compare its neutralization efficacy with the reported efficacy of other antivenom products, i.e., SAIMR polyvalent antivenom (South African Institute for Medical Research, South Africa), Antivipmyn-Africa (Bioclon Institute, Mexico), and VACSERA (The Egyptian Company for Biological Products and Vaccines, Giza, Egypt) [16,17,18]. Of note, SAIMR polyvalent antivenom appeared to be most potent (in neutralizing *N. nivea* venom lethality per gram of antivenom protein), and the logical interpretation is that *N. nivea* venom was used as part of the immunogen cocktail for hyperimmunization during the antivenom manufacturing, in contrast to the other products that lack species-specificity for *N. nivea*. Between Antivipmyn and VAPAV, the former was approximately twice as potent. The immunogens used in the production of both antivenoms include cobra venoms from *N. haje*, *N. melanoleuca* and *N. nigricollis*, whereas Antivipmyn has an additional component from *Naja pallida*. Another antivenom product, VACSERA, was found to be ineffective against *N. nivea* venom, although *N. haje* and *N. nigricollis* venoms were included as the immunogen in its production [18]. The discrepancy in the neutralization efficacy of these antivenoms against *N. nivea* venom could be related to the composition of venom immunogen used by the respective manufacturers or technical factors, e.g., horses’ health conditions in mounting an immune response, immunoglobulin purification method, product purity, and experimental conditions such as the lethal dose (challenge dose) used in the neutralization assay.

Although the commonly adopted venom-antivenom preincubation technique is preferred as a standard method, it should be noted that the *in vivo* interaction between venom and antivenom can be complex. This can be affected by the toxicokinetics of various toxins in the venom, and the pharmacokinetics of different antivenom preparations, thus contributing to variations between studies. Thus, to further verify the *in vivo* cross-neutralization potential of VAPAV, the experimental envenoming and rescue mouse experiment was conducted, mimicking the actual treatment of snakebite where the antivenom is administered as indicated by the sign of neurotoxicity. The *in vivo* neutralization effect of VAPAV, when given as a bolus upon the onset of paralysis in the envenomed mice, was moderate. The antivenom was able to reduce fatality (rescue with VAPAV resulted in 75% survival of the envenomed mice), although the reversal of neurotoxicity was prolonged (close to 24 h) for the envenomed mice to regain normal limb functions. The finding of slow recovery from neurological paralysis echoes the clinical observation of difficulty in reversing the already developed paralysis in patients envenomed by *N. nivea* even with the use of the specific antivenom SAIMR [14]. In contrast, using the same experimental model of envenoming and rescue, previous works from the same laboratory showed that effective antivenoms were able to reverse venom-induced neurotoxicity rapidly, and fully rescue all mice envenomed with *N. naja* and *N. kaouthia* venoms (100% survival) [22,28]. A slow recovery from neurotoxicity indicates that a higher dose of antivenom may be required, or the antivenom may have to be administered repeatedly in order to expedite the clearance of residual toxins and promote recovery. The finding, therefore, revealed the limitation of the heterologous antivenom in cross-neutralizing *N. nivea* venom, and underscored the need for a poly-specific antivenom of high potency that can neutralize the venom effectively. This may be achieved by enriching the venom immunogen formulation with *N. nivea* venom or, more ideally, its fractionated principal neurotoxins. This can be seen in the production of an experimental pan-region, poly-specific antivenom that utilized a diverse toxin repertoire containing venoms and toxins from multiple elapid species [63,64].

## 4. Conclusions

The present study unveiled the venom proteome of *N. nivea* and showed it is dominated by three-finger toxins (3FTx, >80% of total venom proteins), especially cytotoxins/cardiotoxins (~75.6%) and alpha-neurotoxins (~7.4%). The venom proteome lacks PLA_2_—a unique venom phenotype increasingly recognized in the African non-spitting cobras of the *Uraeus* subgenus. The hetero-specific antivenom, VAPAV (an Indian antivenom product manufactured for African use) showed immunoreactivity toward the venoms of *N. nivea* and the phylogenetically related *N. haje* (whose venom was used in VAPAV production), while antivenomics indicated that the venom’s alpha-neurotoxins were immunorecognized by the antivenom with a binding capacity of ~70%. In mice, the antivenom was moderately effective in cross-neutralizing the venom’s lethality. The finding implies the need for further refinement of antivenom production, so that a pan-region antivenom with a higher potency and a broader coverage of snake species can be developed for snakebite envenoming in southern Africa where *N. nivea* envenoming is endemic and prevalent.

## 5. Materials and Methods

### 5.1. Chemicals and Materials

The chemicals and reagents were of analytical grade. Ammonium bicarbonate, dithiothreitol (DTT), iodoacetamide (IAA), Coomassie Brilliant Blue R-250 and phosphate-buffered saline (PBS) were purchased from Sigma-Aldrich (St. Louis, MO, USA). Pierce™ MS grade trypsin protease, Spectra™ Multicolor Broad Range Protein Ladder (10–260 kDa, catalog number: 26634), Bicinchoninic Acid (BCA) Protein Assay kit and trifluoroacetic acid (TFA) were purchased from Thermo Scientific™ Pierce™ (Thermo Fisher Scientific, Waltham, MA, USA). Millipore ZipTip^®^ C_18_ Pipette Tips and HPLC grade acetonitrile (ACN), and LiChrospher^®^ WP 300 RP-18 (5 µm particle size) RP-HPLC column were obtained from Merck (Kenilworth, NJ, USA). Jupiter^®^ 5μm C18 column (300 Å, LC column 250 mm × 4.6 mm) was purchased from Waters (Milford, MA, USA).

### 5.2. Venom and Antivenom

The venoms of *N. nivea* and *N. haje* were supplied by Latoxan (Valence, France). The antivenom used in the present study was VINS African Polyvalent Antivenom (VAPAV; product name: Snake Venom Antiserum—African; batch no.: 07AS16004; expiry date: February 2020; manufacturer: VINS Bioproducts Limited, Hyderabad, India). VAPAV is a sterile preparation containing immunoglobulin fragments F(ab)’_2_ derived from horses hyperimmunized with the venoms of *Naja melanoleuca*, *Naja nigricollis*, *N. haje*, *Dendroaspis polylepis*, *Dendroaspis viridis*, *Dendroaspis jamesoni*, *Bitis gabonica*, *Bitis arietans*, *Echis leucogaster* and *Echis ocellatus*. The lyophilized antivenom was reconstituted in 10 mL ultrapure water prior to use. The antivenom was used before the expiration date.

### 5.3. Animal Supply 

ICR albino mice (20–25 g) used in the preincubation neutralization study were supplied by the Animal Experimental Unit, Faculty of Medicine, University of Malaya. Mice of the same strain and body weight were sourced from the BioLASCO Taiwan Co. Ltd. for the envenoming-rescue study conducted in the National Tsing Hua University, Taiwan. All animal study protocols were referenced to the guidelines provided by the Council for International Organizations of Medical Sciences (CIOMS) [65]. 

### 5.4. Reverse-Phase High-Performance Liquid Chromatography (RP-HPLC)

Three milligrams of *N. nivea* venom were reconstituted in 200 µL ultrapure water, and the supernatant was subjected to LiChrospher^®^ WP300 C-18 reverse-phase fractionation using Shimadzu LC-20 AD HPLC system (Shimadzu, Kyoto, Japan). The C18 column was pre-equilibrated with 0.1% TFA in water (Buffer A), and the sample was separated with 0.1% TFA in ACN (Buffer B) using a linear gradient of 5% B for 10 min, 5–15% B over 20 min, 15–45% B over 120 min and 45–70% B over 20 min at a flow rate of 1 mL/min. The elution of the proteins was monitored at wavelength 215 nm, and fractions were collected manually, lyophilized, and stored at −20 °C until use.

### 5.5. Sodium Dodecyl Sulfate-Polyacrylamide Gel Electrophoresis (SDS-PAGE)

*Naja nivea* venom and the RP-HPLC-collected fractions were reconstituted in ultrapure water and separated by 15% SDS-PAGE under reducing conditions at 100 V for 2 h. Spectra™ Multicolor Broad Range Protein Ladder (10–260 kDa) was used for molecular mass calibration. Gels were stained with Coomassie Brilliant Blue R-250 and scanned using Image ScannerIII Labscan 6.0 (GE Healthcare, Uppsala, Sweden).

### 5.6. In-Solution Tryptic Digestion and Liquid Chromatography-Tandem Mass Spectrometry

RP-HPLC-collected protein fractions (10 µg each) were reduced by dithiothreitol (DTT) and alkylated using iodoacetamide (IAA). MS-grade trypsin (Thermo Fisher Scientific, Waltham, MA, USA) was subsequently used to digest the proteins according to the manufacturer’s protocol. The peptides were then desalted and concentrated using Millipore ZipTip^®^ C18 Pipette Tips. The digested peptides were then reconstituted in 7 µL of 0.1% formic acid in the water and analyzed using nano-electrospray ionization liquid chromatography-tandem mass spectrometry (nano-ESI-LCMS/MS). Agilent 1260 Infinity Nanoflow LC system, coupled with Agilent 6550 Accurate-Mass Q-TOF LC/MS system (Agilent, Santa Clara, CA, USA), was used for the analysis. The samples were loaded to HPLC Large-Capacity Chip Column Zorbax 300-SB-C18 (160 nL enrichment column, 75 µm × 150 mm analytical column and 5 µm particles; Agilent part no. G4240-62010; Agilent, Santa Clara, CA, USA) for peptide separation. The injection volume was 1 μL per sample, and the flow rate was set to 0.4 μL/min, with a linear gradient of 5–70% of elution solvent (0.1% formic acid in 100% acetonitrile). The flow of drying gas was delivered at 11 L/min at 290 °C. The fragmentor voltage was set to 175 V, and the capillary voltage was 1800 V. The mass spectra were obtained using Mass Hunter acquisition software (Agilent, Santa Clara, CA, USA) in MS/MS mode with an MS scan range of 200–3000 *m*/*z* and MS/MS scan range of 50–3200 *m*/*z*. Data were extracted based on MH+ mass range between 50 and 3200 Da and processed with Agilent Spectrum Mill MS Proteomics Workbench software packages version B.04.00. The merged database incorporating non-redundant NCBI databases of Serpentes (taxid: 8570) and an in-house transcripts database was used for peptide matching and protein identification purposes. Carbamidomethylation was specified as a fixed modification and oxidized methionine as a variable modification. The following filters were used for the validation of peptides and proteins: protein score >20 and peptide score >10. Results of protein identification based on ≥2 “distinct peptides” were considered significant.

### 5.7. Estimation of Protein Relative Abundance

The relative abundance of proteins (%) was estimated based on the chromatographic peak area of protein eluted and the mean spectral intensity (MSI) of peptides previously described [25]. The following calculation was adopted:

$$\begin{array}{c} \mathrm{Relative}\;\mathrm{abundance}\;\mathrm{of}\;\mathrm{protein}\;A\;\mathrm{in}\;\mathrm{fraction}\;Z\;\left(\%\right)\\ \;\;\;\;\;\;\;\;\;\;\;\;\;\;\;\;\;\;\;\;=\frac{\mathrm{Mean}\;\mathrm{spectral}\;\mathrm{intensity}\;\mathrm{of}\;\mathrm{protein}\;A\;\mathrm{in}\;\mathrm{fraction}\;Z}{\mathrm{Total}\;\mathrm{mean}\;\mathrm{spectral}\;\mathrm{intensity}\;\mathrm{in}\;\mathrm{HPLC}\;\mathrm{fraction}\;Z}\\ \;\;\;\;\;\;\;\;\;\;\;\;\;\;\;\;\;\;\;\times \;\%\;\mathrm{AUC}\;\mathrm{of}\;\mathrm{HPLC}\;\mathrm{fraction}\;Z \end{array}$$

### 5.8. Immunoreactivity of Antivenom

The immunoreactivity of VAPAV toward the venom antigens of *N. nivea* and *N. haje* were studied with an indirect enzyme-linked immunosorbent assay (indirect ELISA) as previously described [66]. The wells of the 96-well immunoplate were pre-coated with 10 ng of venom antigen at 4 °C overnight. The immunoplate was flicked dry and rinsed with phosphate-buffered saline with 0.5% Tween^®^ 20 (PBST) 4 times to remove the excess antigens. The antivenom was appropriately diluted at 1:100, 1:300, 1:900, 1:2700, 1:8100, and 1:24,300 from a stock concentration of 20 mg/mL. A total of 100 µL of diluted antivenom was then added to each of the antigen-coated wells for incubation at room temperature for 1 h. The incubates were then removed by flicking the immunoplates and washing them with PBST. 100 µL of horseradish peroxidase-conjugated antihorse-IgG (Jackson ImmunoResearch Inc., West Grove, PA, USA) pre-diluted in PBST (1:10,000) was then added into each well, and the incubation took place for 1 h at room temperature. The excess unbound conjugated antibodies were then removed by flicking the immunoplates and washing them 4 times with PBST. Fifty microliters of freshly prepared 3,3′5,5′-tetramethylbenzidine (TMB) substrate solution was subsequently added, and the enzymatic reaction took place in the dark for 10 min at room temperature. The reaction was terminated by adding 50 µL of 12.5% sulfuric acid, and the absorbance was measured at 450 nm using a Tecan i-control™ infinite M1000Pro microplate reader (Tecan, Männedorf, Switzerland). All values were means ± S.E.M. of triplicate experiments. The immunoreactivity was expressed as the half-maximal effective concentrations (EC_50_), interpreted as the antivenom concentration that results in 50% of the venom-antivenom binding reaction.

### 5.9. Antivenomics: Immunorecogniztion of N. nivea Venom Fractions by Antivenom

The ability of VAPAV to immunorecognize *N. nivea* venom fractions was examined using an affinity chromatography approach. Briefly, 1 mL of NHS-activated Sepharose 4 fast flow (GE Healthcare, Danderyd, Sweden) was packed in a column and washed with five matrix volumes (or column volume, CV) of 1 mM ice-cold HCl followed by two CV of coupling buffer (0.2 M NaHCO_3_, 0.5 M NaCl, pH 8.3). The matrix was then incubated with 50 mg of antivenom dissolved in 1 CV of coupling buffer at room temperature for 3 h. After the immobilization of antivenom to the column matrix, the non-reactive NHS matrix groups were blocked with 1 mM ethanolamine at room temperature for 30 min. The column matrix was repeatedly washed six times with 5 CV of low (0.1 M sodium acetate, 0.5 M NaCl, pH 4.0) and high pH buffer (0.5 M ethanolamine, 0.5 M NaCl, pH 8.3) before equilibration with 5 CV of binding buffer (PBS). One milligram of *N. nivea* venom dissolved in 1 mL of binding buffer was loaded to the antivenom-immobilized column matrix and incubated at room temperature for 30 min. Non-retained fractions were collected with 5 CV of PBS, and the immunocaptured venom fractions were eluted with 5 CV of elution buffer (0.1 mM glycine, pH 1.5, neutralized immediately with 1M Tris-HCl, pH 9.0). Both retained, and non-retained fractions were concentrated and subjected to reverse-phase high-performance liquid chromatography (RP-HPLC).

RP-HPLC was performed with a C18 column (300 Å, 250 mm × 4.6 mm particle size, 5 μm pore size; Phenomenex, CA Torrance) using a Shimadzu LC-20AD HPLC system (Shimadzu, Kyoto, Japan). The column was pre-equilibrated with 0.1 % TFA in ultrapure water, and eluted with a linear gradient of 0.1% TFA in acetonitrile (Buffer B) at 2% B for 5 min, 2–10% B over 2 min, 10–16% B over 6 min, 16–28% B over 2 min, 28–65% B over 37 min and 65–80% B over 5 min, with a flow rate of 0.3 mL/min. The venom fractions were monitored at a wavelength of 280 nm. The degree of VAPAV immunorecognition toward a venom fraction was estimated by the chromatographic peak area under the curve (AUC) using OriginLab 8.0 software (OriginLab Corporation, Northampton, UK). The immunorecognition was expressed as the percentage of proteins bound by the antivenom-coated affinity column (regarded as antivenom-treated venom) in comparison to that of the untreated venom profiled by the RP-HPLC under the same conditions.

### 5.10. Venom Lethality and Neutralization by Antivenom

A hundred microliters of the venom in various doses were injected intravenously into ICR mice through the caudal vein (20–25 g, *n* = 4 per dose). The survival ratio at each venom dose was recorded at 24 h post-injection. In the lethality neutralization assay, a challenge dose of the venom (5 × LD_50_) was pre-incubated with various dilutions of antivenom in a total volume of 200 µL at 37 °C for 30 min, followed by intravenous injection into the mice through the caudal vein (20–25 g, *n* = 4 per dose). The survival ratio of mice was recorded at 24 h post-injection. The venom intravenous median lethal dose (LD_50_), antivenom median effective dose (ED_50_) and the 95% confidence intervals (C.I.) were calculated by the Probit analysis method [67], using BioStat 2009 analysis software (AnalystSoft Inc., Walnut, CA, USA). The neutralizing capacity was also expressed as potency (P), defined as the amount of venom completely neutralized by one mL of antivenom (mg/mL), calculated as previously described [68,69]. For comparison purposes, the potency (P) was further divided by the antivenom protein concentration to obtain normalized potency (n-P), defined as the amount of venom neutralized by one gram of antivenom protein (mg/g).

### 5.11. Experimental Envenoming and Rescue Experiment

The experiment mimics the natural setting of snakebite and antivenom treatment. It was conducted in an envenoming and rescue model in mice as previously described [22,70]. The subcutaneous LD_50_ of *N. nivea* venom was first established by injecting the venom at various doses into the loose skin over the neck of the mice (20–25 g, *n* = 4 per dose). The number of deaths of the mice at each dose was recorded at 24 h. In the envenoming and rescue model, a challenge dose of venom (5 × *s.c*. LD_50_) dissolved in 50 µL saline was inoculated subcutaneously into the mice (20–25 g, *n* = 8). The mice were monitored closely for the development of neurological signs. In the rescue group, the antivenom (VAPAV) was administered as a bolus of 250 µL intravenously (via the caudal vein) upon the onset of neurotoxicity indicated by posterior limb paralysis. Mice in the control (untreated) group (20–25 g, *n* = 8) received 250 µL of saline instead upon the onset of neurotoxicity. The progress of neurotoxicity (deterioration or reversal) in the mice was closely monitored over 24 h. All mice were allowed free access to food and water *ad libitum* throughout the experiment.

## Figures and Tables

**Table 1:** Proteins identified from *N. nivea* venom fractions profiled by C18 reverse-phase high-performance liquid chromatography and nano-ESI-LCMS/MS.

Protein Name	Database Accession ^a^	Species	MS Search Score ^b^	R.A. (%) ^c^
**Fraction 1**				
Short neurotoxin 2	P01422	*Naja annulifera*	38.46	1.58
**Fraction 2**				
Short neurotoxin 4	P01421	*Naja annulifera*	123.04	1.88
Long neurotoxin 1	P01390	*Naja nivea*	89.87	0.09
Weak toxin S4C11	P01400	*Naja melanoleuca*	46.32	0.04
**Fraction 3**				
Long neurotoxin 1	P01390	*Naja nivea*	97.38	2.85
Short neurotoxin 4	P01421	*Naja annulifera*	53.91	0.07
Weak toxin CM-11	P01401	*Naja haje haje*	44.62	0.17
**Fraction 4**				
Long neurotoxin 1	P01390	*Naja nivea*	114.03	0.02
Weak toxin CM-13b	P01399	*Naja annulifera*	97.34	0.42
Short neurotoxin 4	P01421	*Naja annulifera*	34.64	0.08
**Fraction 5**				
Kunitz-type serine protease inhibitor 2	P00986	*Naja nivea*	141.56	1.16
Long neurotoxin 1	P01390	*Naja nivea*	114.03	0.34
Weak toxin CM-10	P25680	*Naja nivea*	57.78	0.91
**Fraction 6**				
Long neurotoxin 1	P01390	*Naja nivea*	102.63	0.32
Kunitz-type serine protease inhibitor 2	P00986	*Naja nivea*	82.24	0.07
Weak toxin CM-13b	P01399	*Naja annulifera*	34.6	0.04
**Fraction 7**				
Cytotoxin 1	P01456	*Naja nivea*	210.7	25.63
Cytotoxin 2	P01462	*Naja annulifera*	65.61	0.74
Long neurotoxin 1	P01390	*Naja nivea*	58.24	0.16
**Fraction 8**				
Cytotoxin 1	P01456	*Naja nivea*	102.98	0.67
Cytotoxin 8	P01460	*Naja annulifera*	74.54	1.35
Cytotoxin 2	P01463	*Naja nivea*	68.3	0.26
Cytotoxin 3	P01458	*Naja nivea*	51.41	0.40
**Fraction 9**				
Cytotoxin 2	P01462	*Naja annulifera*	131.23	4.41
Cytotoxin 7	P01466	*Naja annulifera*	116.39	5.03
Cytotoxin 8	P01460	*Naja annulifera*	99.86	6.97
Cytotoxin 10	P01453	*Naja annulifera*	88.36	2.10
Cytotoxin 3	P01458	*Naja nivea*	66.72	0.25
Cytotoxin 1	P01456	*Naja nivea*	61.69	3.04
Venom nerve growth factor 2	Q5YF89	*Naja sputatrix*	34.62	0.09
**Fraction 10**				
Cytotoxin 2	P01463	*Naja nivea*	161.32	1.91
Cytotoxin 3	P01458	*Naja nivea*	164.72	9.68
Cytotoxin 7	P01466	*Naja annulifera*	97.35	2.92
Cytotoxin 10	P01453	*Naja annulifera*	64.96	2.41
Cytotoxin 8	P01460	*Naja annulifera*	52.47	4.93
Venom nerve growth factor 2	CL429.Contig1_NnSL	*Naja naja*	42.38	1.12
**Fraction 11**				
Cytotoxin homolog	P14541	*Naja kaouthia*	98.04	1.78
Cytotoxin 2	P01463	*Naja nivea*	72.38	0.43
Cytotoxin 1	P01456	*Naja nivea*	42.36	0.07
Cytotoxin 3	P01459	*Naja annulifera*	40.31	0.21
Cytotoxin 1	P01468	*Naja pallida*	34.49	0.16
Venom nerve growth factor 2	Q5YF89	*Naja sputatrix*	32.84	0.01
**Fraction 12**				
Thaicobrin	P82885	*Naja kaouthia*	89.06	0.04
Cytotoxin 3	P01458	*Naja nivea*	87.75	0.03
Cytotoxin 2	P01463	*Naja nivea*	77.44	0.05
Cytotoxin 1	P01456	*Naja nivea*	74.18	0.02
Cytotoxin 1	P01468	*Naja pallida*	61.79	0.03
Cytotoxin 8	P01460	*Naja annulifera*	61.72	0.12
Cytotoxin 10	P01453	*Naja annulifera*	58.41	0.02
Venom nerve growth factor 2	Q5YF89	*Naja sputatrix*	81.11	<0.01
Cytotoxin homolog	P14541	*Naja kaouthia*	61.23	0.01
Cytotoxin 11	P62394	*Naja haje haje*	44.22	0.01
**Fraction 13**				
Cysteine-rich venom protein natrin-2	Q7ZZN8	*Naja atra*	88.14	0.44
Cysteine-rich venom protein latisemin	Q8JI38	*Laticauda semifasciata*	41.66	0.16
Venom nerve growth factor 2	Q5YF89	*Naja sputatrix*	72.47	0.01
Zinc metalloproteinase-disintegrin atragin	CL626.Contig4_NsM	*Naja sumatrana*	35.12	0.08
**Fraction 14**				
Natrin-1	CL85.Contig1_NnSL	*Naja naja*	139.79	1.41
Cysteine-rich venom protein natrin-1	Q7T1K6	*Naja atra*	134.04	1.41
Zinc metalloproteinase-disintegrin cobrin	CL2966.Contig3_NnSL	*Naja naja*	67.86	0.85
Scutellatease-1	CL2215.Contig1_HsM	*Enhydrina schistosa*	38.33	0.31
Cysteine-rich secretory protein Pg-CRP	F2Q6F6	*Cerrophidion godmani*	34.78	0.04
SVMP-Aca-4	R4G2D3	*Acanthophis wellsi*	29.96	0.03
**Fraction 15**				
Natrin-1	CL85.Contig1_NnSL	*Naja naja*	72.73	0.10
Microlepidotease-1	B5KFV6	*Oxyuranus microlepidotus*	52.23	0.15
Metalloproteinase (Type III) 1	U3EPC7	*Micrurus fulvius*	41.16	0.15
Zinc metalloproteinase-disintegrin-like atragin	CL444.Contig1_NsM	*Naja sumatrana*	53.63	0.03
Hemorrhagic metalloproteinase-disintegrin-like kaouthiagin	P82942	*Naja kaouthia*	49.13	0.21
SVMP-Aca-4	R4G2D3	*Acanthophis wellsi*	44.77	0.01
Zinc metalloproteinase-disintegrin-like atrase-A	D5LMJ3	*Naja atra*	48.07	1.49
Zinc metalloproteinase-disintegrin cobrin	CL2966.Contig3_NnSL	*Naja naja*	47.8	1.52
Zinc metalloproteinase-disintegrin cobrin	CL7366.Contig1_OhM	*Ophiophagus hannah*	30.86	0.03
**Fraction 16**				
Phosphodiesterase 1	Unigene5869_NsM	*Naja sumatrana*	167.77	0.15
Phosphodiesterase	A0A194ARD7	*Micrurus tener*	129.25	0.30
Phosphodiesterase 1	CL4383.Contig2_OhM	*Ophiophagus hannah*	89.99	0.54
Snake venom 5′-nucleotidase	CL3600.Contig1_NsM2	*Naja sumatrana*	119.17	0.38
Snake venom 5′-nucleotidase	CL4180.Contig1_OhM	*Ophiophagus hannah*	108.51	0.36
Zinc metalloproteinase-disintegrin-like atragin	CL444.Contig1_NsM	*Naja sumatrana*	78.8	0.62
Zinc metalloproteinase-disintegrin-like atragin	D3TTC2	*Naja atra*	45.45	0.21
Zinc metalloproteinase-disintegrin atragin	CL2051.Contig1_NkM	*Naja kaouthia*	29.61	0.33
**Fraction 17**				
Cobra venom factor	Unigene370_NsM2	*Naja sumatrana*	268.75	0.03
Cobra venom factor	CL4560.Contig1_NsM	*Naja sumatrana*	267	0.04
Cobra venom factor	Q91132	*Naja kaouthia*	136.91	0.13
L-amino-acid oxidase	CL4047.Contig1_NsM2	*Naja sumatrana*	202.09	0.07
L-amino-acid oxidase	A8QL58	*Naja atra*	187.13	0.06
L-amino acid oxidase	A8QL51	*Bungarus multicinctus*	67.97	0.15
L-amino-acid oxidase	CL2322.Contig1_HhSL	*Hypnale hypnale*	36.77	0.20
Acetylcholinesterase	CL4231.Contig1_NsM	*Naja sumatrana*	114.16	0.05
Acetylcholinesterase	Unigene16279_OhM	*Ophiophagus hannah*	85.55	0.05
Snake venom 5′-nucleotidase	CL3600.Contig1_NsM2	*Naja sumatrana*	77.5	0.01
Snake venom metalloproteinase-disintegrin-like mocarhagin	Q10749	*Naja mossambica*	65.43	0.24
SVMP-Hop-45	R4G2Y9	*Hoplocephalus bungaroides*	36.28	0.38
Zinc metalloproteinase-disintegrin cobrin	CL115.Contig9_NkT	*Naja kaouthia*	30.06	0.01
Zinc metalloproteinase-disintegrin atragin	CL626.Contig4_NsM2	*Naja sumatrana*	54.02	0.02
Zinc metalloproteinase mocarhagin	Unigene25077_NnSL	*Naja naja*	32.68	0.04
Zinc metalloproteinase-disintegrin-like VLAIP-A	Q4VM08	*Macrovipera lebetina*	30.22	0.11
Phosphodiesterase 1	unigene5869_NsM	*Naja sumatrana*	27.96	<0.01

^a^ Protein codes with the prefixes “CL” and “Unigene” were derived from the in-house transcriptome database. ^b^ MS search score refers to the protein score based on peptides matched to databases during the protein identification process by LCMS/MS. A score of ≥20 was set as a filter for protein identification in this study. ^c^ Protein relative abundance was determined as the percentage of total venom proteins.

**Table 2:** Venom proteome of *N. nivea* according to protein families and relative abundance.

Protein Family	Protein Name	Accession ^a^	Species	R.A. (%) ^b^
**Three-finger toxins (3FTX)**				**84.62**
	**Short neurotoxin (SNTX)**			**3.61**
	Short neurotoxin 2	P01422	*Naja annulifera*	1.58
	Short neurotoxin 4	P01421	*Naja annulifera*	2.03
	**Long neurotoxin (LNTX)**			**3.78**
	Long neurotoxin 1	P01390	*Naja nivea*	3.78
	**Weak neurotoxin (WTX)**			**1.58**
	Weak toxin CM-13b	P01399	*Naja annulifera*	0.45
	Weak toxin S4C11	P01400	*Naja melanoleuca*	0.04
	Weak toxin CM-11	P01401	*Naja haje haje*	0.17
	Weak toxin CM-10	P25680	*Naja nivea*	0.91
	**Cytotoxin/cardiotoxin (CTX)**			**75.65**
	Cytotoxin 10	P01453	*Naja annulifera*	4.53
	Cytotoxin 1	P01456	*Naja nivea*	29.43
	Cytotoxin 3	P01458	*Naja nivea*	10.36
	Cytotoxin 3	P01459	*Naja annulifera*	0.21
	Cytotoxin 8	P01460	*Naja annulifera*	13.37
	Cytotoxin 2	P01462	*Naja annulifera*	5.15
	Cytotoxin 2	P01463	*Naja nivea*	2.65
	Cytotoxin 7	P01466	*Naja annulifera*	7.95
	Cytotoxin 1	P01468	*Naja pallida*	0.20
	Cytotoxin homolog	P14541	*Naja kaouthia*	1.79
	Cytotoxin 11	P62394	*Naja haje haje*	<0.01
**Snake venom metalloproteinase (SVMP)**				**6.79**
	microlepidotease-1	B5KFV6	*Oxyuranus microlepidotus*	0.15
	Zinc metalloproteinase-disintegrin cobrin	CL115.Contig9_NkT	*Naja kaouthia*	0.01
	Zinc metalloproteinase-disintegrin atragin	CL2051.Contig1_NkM	*Naja kaouthia*	0.33
	Scutellatease-1	CL2215.Contig1_HsM	*Hydrophis schistosus*	0.30
	Zinc metalloproteinase-disintegrin cobrin	CL2966.Contig3_NnSL	*Naja naja*	2.37
	Zinc metalloproteinase-disintegrin-like atragin	CL444.Contig1_NsM	*Naja sumatrana*	0.64
	Zinc metalloproteinase-disintegrin atragin	CL626.Contig4_NsM2	*Naja sumatrana*	0.10
	Zinc metalloproteinase-disintegrin cobrin	CL7366.Contig1_OhM	*Ophiophagus hannah*	0.03
	Zinc metalloproteinase-disintegrin-like atragin	D3TTC2	*Naja atra*	0.21
	Zinc metalloproteinase-disintegrin-like atrase-A	D5LMJ3	*Naja atra*	1.49
	Hemorrhagic metalloproteinase-disintegrin-like kaouthiagin	P82942	*Naja kaouthia*	0.21
	Snake venom metalloproteinase-disintegrin-like mocarhagin	Q10749	*Naja mossambica*	0.24
	Zinc metalloproteinase-disintegrin-like VLAIP-A	Q4VM08	*Macrovipera lebetina*	0.11
	SVMP-Aca-4	R4G2D3	*Acanthophis wellsi*	0.04
	SVMP-Hop-45	R4G2Y9	*Hoplocephalus bungaroides*	0.38
	Metalloproteinase (Type III) 1	U3EPC7	*Micrurus fulvius*	0.15
	Zinc metalloproteinase mocarhagin	unigene25077_NnSL	*Naja naja*	0.04
**Cysteine-rich secretory protein (CRISP)**				**3.56**
	Natrin-1	CL85.Contig1_NnSL	*Naja naja*	1.51
	Cysteine-rich venom protein ophanin	Q7ZT98	*Ophiophagus hannah*	0.04
	Cysteine-rich venom protein natrin-1	Q7T1K6	*Naja atra*	1.41
	Cysteine-rich venom protein natrin-2	Q7ZZN8	*Naja atra*	0.44
	Cysteine-rich venom protein latisemin	Q8JI38	*Laticauda semifasciata*	0.16
**Kunitz-type serine protease inhibitor (KSPI)**				**1.24**
	Kunitz-type serine protease inhibitor 2	P00986	*Naja nivea*	1.24
**Nerve growth factor (NGF)**				**1.22**
	Venom nerve growth factor 2	CL429.Contig1_NnSL	*Naja naja*	1.12
	Venom nerve growth factor 2	Q5YF89	*Naja sputatrix*	0.10
**Phosphodiesterase (PDE)**				**1.00**
	Phosphodiesterase	A0A194ARD7	*Micrurus tener*	0.30
	Phosphodiesterase 1	CL4383.Contig2_OhM	*Ophiophagus hannah*	0.54
	Phosphodiesterase 1	unigene5869_NsM	*Naja sumatrana*	0.15
**5′-nucleotidase (5′-NUC)**				**0.75**
	Snake venom 5′-nucleotidase	CL3600.Contig1_NsM2	*Naja sumatrana*	0.39
	Snake venom 5′-nucleotidase	CL4180.Contig1_OhM	*Ophiophagus hannah*	0.36
**L-amino acid oxidase (LAAO)**				**0.48**
	L-amino-acid oxidase	CL4047.Contig1_NsM2	*Naja sumatrana*	0.07
	L-amino-acid oxidase	A8QL58	*Naja atra*	0.06
	L-amino acid oxidase	A8QL51	*Bungarus multicinctus*	0.15
	L-amino-acid oxidase-like	A0A6J1W8Y3	*Notechis scutatus*	0.20
**Cobra venom factor (CVF)**				**0.20**
	Cobra venom factor	unigene370_NsM2	*Naja sumatrana*	0.03
	Cobra venom factor	CL4560.Contig1_NsM	*Naja sumatrana*	0.04
	Cobra venom factor	Q91132	*Naja kaouthia*	0.13
**Acetylcholinesterase (AChE)**				**0.10**
	Acetylcholinesterase NS2	CL4231.Contig1_NsM	*Naja sumatrana*	0.05
	Acetylcholinesterase	unigene16279_OhM	*Ophiophagus hannah*	0.05
**Vespryn (VES)**				**0.04**
	Thaicobrin	P82885	*Naja kaouthia*	0.04

^a^ Accession codes with the suffix “_xxxx” were proteins identified based on tryptic peptides matched to sequences from an in-house transcript database. ^b^ RA: Relative abundance is represented as the percentage of total venom proteins.

**Table 3:** Immunorecognition of *N. nivea* venom fractions by VAPAV.

Number of Protein Fractions ^a^	Immunorecognition Capacity of VAPAV ^b^ (%)
1	67.0
2, 3	70.2
4	24.8
5	22.3
6	22.9
7	25.5
8	24.4
9, 10, 11	31.3
12	58.8

^a^ Numbering based on the reverse-phase HPLC profiles of *N. nivea* venom (see Figure 4). ^b^ Percentage indicates the ratio of proteins in each fraction bound or immunoretained by the VAPAV-immobilized affinity column.

**Table 4:** Lethality of *N. nivea* venom and its neutralization by antivenoms used in Africa.

*i.v.* LD_50_ ^a^ (µg/g)	*s.c.* LD_50_ ^a^ (µg/g)	Antivenom	Antivenom Protein Concentration (mg/mL)	Challenge Dose	ED_50_ ^b^ (µL)	ER_50_ ^c^ (mg/mL)	Potency ^d^ (mg/mL)	Normalized Potency ^e^ (n-P)	Reference
1.11 (0.73–1.69)	2.42 (2.10–2.79)	VAPAV	130.68 ± 2.83 ^#^	2.5	74.61	0.86 (0.61–1.20)	0.51	3.90	Current study
0.84 (0.57–1.04)	-	SAIMR	118.4	2.0	17.81	1.70 (1.49–1.94)	0.94	7.94	[16]
0.45 (0.43–0.47)	-	Antivipmyn-Africa	32	3.0	57.11	0.47 (0.47–0.48)	0.31	9.69	[17]
3.55 (95% CI: NA)	-	VACSERA	NA	NA	Not effective				[18]

Abbreviation: *i.v.*, intravenous; *s.c.*, subcutaneous; LD_50_, median lethal dose; ED_50_, median effective dose; ER_50_, median effective ratio; NA, not available. ^a^ LD_50_, the dose of venom (μg) per gram of mouse body weight at which 50% of mice died. ^b^ ED_50_, the dose of antivenom (μL) at which 50% of mice survived. ^c^ ER_50_, the ratio of venom (mg) to the volume of antivenom (ml) at which 50% of mice survived. ^d^ Potency, the amount of venom (mg) completely neutralized per ml antivenom (mg/mL). ^e^ Normalized potency, the amount of venom (mg) completely neutralized per g antivenom protein (mg/g). ^#^ Protein concentration of VAPAV is as previously reported for the same sample [19].

## Data Availability

The mass spectrometry proteomics data have been deposited to the ProteomeXchange Consortium (http://proteomecentral.proteomexchange.org) via the iProX partner repository (http://www.iprox.org) [71] with the dataset identifier PXD028220 (data depository date: 7 September 2021).

## Supplementary Materials

The following supporting information can be downloaded at: https://www.mdpi.com/article/10.3390/toxins14120860/s1, Supplementary File S1: Mass spectrometric data and peptide sequences of protein identified from the *Naja nivea* venom fractions using nano-ESI-LCMS/MS.

## References

[1] WHO (2016). Guidelines for the Management of Snakebites.

[2] Gutiérrez J.M., Calvete J.J., Habib A.G., Harrison R.A., Williams D.J., Warrell D.A. (2017). Snakebite envenoming. Nat. Rev. Dis. Prim..

[3] Chippaux J.-P., Massougbodji A., Habib A.G. (2019). The WHO strategy for prevention and control of snakebite envenoming: A sub-Saharan Africa plan. J. Venom. Anim. Toxins Incl. Trop. Dis..

[4] Kasturiratne A., Wickremasinghe A.R., de Silva N., Gunawardena N.K., Pathmeswaran A., Premaratna R., Savioli L., Lalloo D.G., de Silva H.J. (2008). The global burden of snakebite: A literature analysis and modelling based on regional estimates of envenoming and deaths. PLoS Med..

[5] Halilu S., Iliyasu G., Hamza M., Chippaux J.-P., Kuznik A., Habib A.G. (2019). Snakebite burden in Sub-Saharan Africa: Estimates from 41 countries. Toxicon Off. J. Int. Soc. Toxinology.

[6] Benjamin J.M., Abo B.N., Brandehoff N. (2020). Review Article: Snake Envenomation in Africa. Curr. Trop. Med. Rep..

[7] Wallach V., Wüster W., Broadley G.D. (2009). In Praise Of Subgenera: Taxonomic Status Of Cobras Of The Genus *Naja* Laurenti (Serpentes: Elapidae). Zootaxa.

[8] Broadley D.G., Wüster W. (2004). A review of the southern African ‘non-spitting’ cobras (Serpentes: Elapidae: *Naja*). Afr. J. Herpetol..

[9] World Health Organization (2010). Guidelines for the Prevention and Clinical Management of Snakebite in Africa.

[10] Christensen P.A. (1953). Problems of antivenene standardization revealed by the flocculation reaction. Bull. World Health Organ.

[11] Hokama Y., Iwanaga S., Tatsuki T., Suzuki T. (1976). Snake venom proteinase inhibitors. III. Isolation of five polypeptide inhibitors from the venoms of *Hemachatus haemachatus* (Ringhal’s corbra) and *Naja nivea* (Cape cobra) and the complete amino acid sequences of two of them. J. Biochem..

[12] Joubert F.J., Taljaard N. (1980). Snake venoms. The amino acid sequences of two Melanoleuca-type toxins. Hoppe. Seylers Z. Physiol. Chem..

[13] Botes D.P., Viljoen C.C. (1976). The amino acid sequence of three non-curarimimetic toxins from *Naja nivea* venom. Biochim. Et Biophys. Acta (BBA)—Protein Struct..

[14] Blaylock R.S., Lichtman A.R., Potgieter P.D. (1985). Clinical manifestations of Cape cobra (*Naja nivea*) bites. A report of 2 cases. South Afr. Med. J. Suid-Afrik. Tydskr. Vir Geneeskd..

[15] Potet J., Smith J., McIver L. (2019). Reviewing evidence of the clinical effectiveness of commercially available antivenoms in sub-Saharan Africa identifies the need for a multi-centre, multi-antivenom clinical trial. PLoS Negl. Trop. Dis..

[16] Whiteley G., Casewell N.R., Pla D., Quesada-Bernat S., Logan R.A.E., Bolton F.M.S., Wagstaff S.C., Gutiérrez J.M., Calvete J.J., Harrison R.A. (2019). Defining the pathogenic threat of envenoming by South African shield-nosed and coral snakes (genus *Aspidelaps*), and revealing the likely efficacy of available antivenom. J. Proteom..

[17] Ramos-Cerrillo B., de Roodt A.R., Chippaux J.P., Olguín L., Casasola A., Guzmán G., Paniagua-Solís J., Alagón A., Stock R.P. (2008). Characterization of a new polyvalent antivenom (Antivipmyn Africa) against African vipers and elapids. Toxicon Off. J. Int. Soc. Toxinology.

[18] Seddik S.S., Wanas S., Helmy M.H., Hashem M. (2002). Cross neutralization of dangerous snake venoms from Africa and the Middle East using the VACSERA polyvalent antivenom. Egyptian Organization for Biological Products & Vaccines. J. Nat. Toxins.

[19] Wong K.Y., Tan K.Y., Tan N.H., Tan C.H. (2021). A Neurotoxic Snake Venom without Phospholipase A_2_: Proteomics and Cross-Neutralization of the Venom from Senegalese Cobra, *Naja senegalensis* (Subgenus: *Uraeus*). Toxins.

[20] Botes D.P. (1971). Snake venom toxins. The amino acid sequences of toxins alpha and beta from *Naja nivea* venom and the disulfide bonds of toxin alpha. J. Biol. Chem..

[21] Palasuberniam P., Chan Y.W., Tan K.Y., Tan C.H. (2021). Snake Venom Proteomics of Samar Cobra (*Naja samarensis*) from the Southern Philippines: Short Alpha-Neurotoxins as the Dominant Lethal Component Weakly Cross-Neutralized by the Philippine Cobra Antivenom. Front. Pharm..

[22] Wong K.Y., Tan C.H., Tan N.H. (2016). Venom and Purified Toxins of the Spectacled Cobra (*Naja naja*) from Pakistan: Insights into Toxicity and Antivenom Neutralization. Am. J. Trop. Med. Hyg..

[23] Tan K.Y., Tan C.H., Fung S.Y., Tan N.H. (2016). Neutralization of the Principal Toxins from the Venoms of Thai *Naja kaouthia* and Malaysian *Hydrophis schistosus*: Insights into Toxin-Specific Neutralization by Two Different Antivenoms. Toxins.

[24] Loots J.M., Meij H.S., Meyer B.J. (1973). Effects of *Naja nivea* venom on nerve, cardiac and skeletal muscle activity of the frog. Br. J. Pharmacol..

[25] Tan C.H., Wong K.Y., Chong H.P., Tan N.H., Tan K.Y. (2019). Proteomic insights into short neurotoxin-driven, highly neurotoxic venom of Philippine cobra (*Naja philippinensis*) and toxicity correlation of cobra envenomation in Asia. J. Proteom..

[26] Wong K.Y., Tan K.Y., Tan N.H., Gnanathasan C.A., Tan C.H. (2021). Elucidating the Venom Diversity in Sri Lankan Spectacled Cobra (*Naja naja*) through *De Novo* Venom Gland Transcriptomics, Venom Proteomics and Toxicity Neutralization. Toxins.

[27] Tan C.H., Tan K.Y., Wong K.Y., Tan N.H., Chong H.P. (2022). Equatorial Spitting Cobra (*Naja sumatrana*) from Malaysia (Negeri Sembilan and Penang), Southern Thailand, and Sumatra: Comparative Venom Proteomics, Immunoreactivity and Cross-Neutralization by Antivenom. Toxins.

[28] Tan K.Y., Tan C.H., Sim S.M., Fung S.Y., Tan N.H. (2016). Geographical venom variations of the Southeast Asian monocled cobra (*Naja kaouthia*): Venom-induced neuromuscular depression and antivenom neutralization. Comp. Biochem. Physiol. Toxicol. Pharmacol. CBP.

[29] Lauridsen L.P., Laustsen A.H., Lomonte B., Gutierrez J.M. (2017). Exploring the venom of the forest cobra snake: Toxicovenomics and antivenom profiling of *Naja melanoleuca*. J. Proteom..

[30] Petras D., Sanz L., Segura A., Herrera M., Villalta M., Solano D., Vargas M., Leon G., Warrell D.A., Theakston R.D. (2011). Snake venomics of African spitting cobras: Toxin composition and assessment of congeneric cross-reactivity of the pan-African EchiTAb-Plus-ICP antivenom by antivenomics and neutralization approaches. J. Proteome. Res..

[31] Tan K.Y., Wong K.Y., Tan N.H., Tan C.H. (2020). Quantitative proteomics of *Naja annulifera* (sub-Saharan snouted cobra) venom and neutralization activities of two antivenoms in Africa. Int. J. Biol. Macromol..

[32] Marais J. (2004). A Complete Guide to the Snakes of Southern Africa.

[33] Nirthanan S., Gopalakrishnakone P., Gwee M.C., Khoo H.E., Kini R.M. (2003). Non-conventional toxins from Elapid venoms. Toxicon Off. J. Int. Soc. Toxinology.

[34] Lyukmanova E.N., Shenkarev Z.O., Shulepko M.A., Paramonov A.S., Chugunov A.O., Janickova H., Dolejsi E., Dolezal V., Utkin Y.N., Tsetlin V.I. (2015). Structural Insight into Specificity of Interactions between Nonconventional Three-finger Weak Toxin from *Naja kaouthia* (WTX) and Muscarinic Acetylcholine Receptors. J. Biol. Chem..

[35] Leong P.K., Fung S.Y., Tan C.H., Sim S.M., Tan N.H. (2015). Immunological cross-reactivity and neutralization of the principal toxins of *Naja sumatrana* and related cobra venoms by a Thai polyvalent antivenom (Neuro Polyvalent Snake Antivenom). Acta Trop..

[36] Condrea E., Devries A., Mager J. (1964). Hemolysis and splitting of human erythrocyte phospholipids by snake venoms. Biochim. Et Biophys. Acta.

[37] Gasanov S.E., Alsarraj M.A., Gasanov N.E., Rael E.D. (1997). Cobra venom cytotoxin free of phospholipase A_2_ and its effect on model membranes and T leukemia cells. J. Membr. Biol..

[38] Dubovskii P.V., Lesovoy D.M., Dubinnyi M.A., Konshina A.G., Utkin Y.N., Efremov R.G., Arseniev A.S. (2005). Interaction of three-finger toxins with phospholipid membranes: Comparison of S- and P-type cytotoxins. Biochem. J..

[39] Chien K.Y., Chiang C.M., Hseu Y.C., Vyas A.A., Rule G.S., Wu W. (1994). Two distinct types of cardiotoxin as revealed by the structure and activity relationship of their interaction with zwitterionic phospholipid dispersions. J. Biol. Chem..

[40] Chong H.P., Tan K.Y., Tan C.H. (2020). Cytotoxicity of snake venoms and cytotoxins from two Southeast Asian cobras (*Naja sumatrana*, *Naja kaouthia*): Exploration of anticancer potential, selectivity, and cell death mechanism. Front. Mol. Biosci..

[41] Epstein D. (1930). The pharmacology of the venom of the cape cobra. Q. J. Exp. Physiol..

[42] Sudulagunta S.R., Sodalagunta M.B., Khorram H., Sepehrar M., Noroozpour Z. (2015). Case Report Cardiotoxicity and respiratory failure due to Cobra bite. Sch. J. Appl. Med. Sci..

[43] Senthilkumaran S., Meenakshisundaram R., Thirumalaikolundusubramanian P., Menezes R.G. (2012). Cardiac toxicity following cobra envenomation. Clin. Toxicol..

[44] Silva de França F., Villas-Boas I.M., Cogliati B., Woodruff T.M., Reis E.d.S., Lambris J.D., Tambourgi D.V. (2021). C5a-C5aR1 axis activation drives envenomation immunopathology by the snake *Naja annulifera*. Front. Immunol..

[45] Yamazaki Y., Hyodo F., Morita T. (2003). Wide distribution of cysteine-rich secretory proteins in snake venoms: Isolation and cloning of novel snake venom cysteine-rich secretory proteins. Arch. Biochem. Biophys..

[46] Tadokoro T., Modahl C.M., Maenaka K., Aoki-Shioi N. (2020). Cysteine-Rich Secretory Proteins (CRISPs) From Venomous Snakes: An Overview of the Functional Diversity in A Large and Underappreciated Superfamily. Toxins.

[47] Mukherjee A.K., Mackessy S.P., Dutta S. (2014). Characterization of a Kunitz-type protease inhibitor peptide (Rusvikunin) purified from *Daboia russelii russelii* venom. Int. J. Biol. Macromol..

[48] Earl S.T.H., Richards R., Johnson L.A., Flight S., Anderson S., Liao A., de Jersey J., Masci P.P., Lavin M.F. (2012). Identification and characterisation of Kunitz-type plasma kallikrein inhibitors unique to *Oxyuranus* sp. snake venoms. Biochimie.

[49] Wijeyewickrema L.C., Gardiner E.E., Gladigau E.L., Berndt M.C., Andrews R.K. (2010). Nerve growth factor inhibits metalloproteinase-disintegrins and blocks ectodomain shedding of platelet glycoprotein VI. J. Biol. Chem..

[50] Aird S.D. (2002). Ophidian envenomation strategies and the role of purines. Toxicon Off. J. Int. Soc. Toxinology.

[51] Aird S.D. (2005). Taxonomic distribution and quantitative analysis of free purine and pyrimidine nucleosides in snake venoms. Comp. Biochem. Physiology. Part B Biochem. Mol. Biol..

[52] Vogel C.-W., Bredehorst R., Fritzinger D.C., Grunwald T., Ziegelmüller P., Kock M.A., Singh B.R., Tu A.T. (1996). Structure and Function of Cobra Venom Factor, the Complement-Activating Protein in Cobra Venom. Natural Toxins 2: Structure, Mechanism of Action, and Detection.

[53] Vogel C.-W., Fritzinger D.C., Inagaki H., Vogel C.-W., Mukherjee A.K., Rahmy T.R. (2017). Cobra Venom Factor: The Unique Component of Cobra Venom That Activates the Complement System. Snake Venoms.

[54] Paloschi M.V., Boeno C.N., Lopes J.A., Rego C.M.A., Silva M.D.S., Santana H.M., Serrath S.N., Ikenohuchi Y.J., Farias B.J.C., Felipin K.P. (2022). Reactive oxygen species-dependent-NLRP3 inflammasome activation in human neutrophils induced by l-amino acid oxidase derived from *Calloselasma rhodostoma* venom. Life Sci..

[55] Cousin X., Bon C. (1999). Acetylcholinesterase from snake venom as a model for its nerve and muscle counterpart. J. Nat. Toxins.

[56] Pung Y.F., Kumar S.V., Rajagopalan N., Fry B.G., Kumar P.P., Kini R.M. (2006). Ohanin, a novel protein from king cobra venom: Its cDNA and genomic organization. Gene.

[57] Tan C.H., Wong K.Y., Tan N.H., Ng T.S., Tan K.Y. (2019). Distinctive Distribution of Secretory Phospholipases A_2_ in the Venoms of Afro-Asian Cobras (Subgenus: *Naja*, *Afronaja*, *Boulengerina* and *Uraeus*). Toxins.

[58] Malih I., Rusmili A.M.R., Tee T.Y., Saile R., Ghalim N., Othman I. (2014). Proteomic analysis of Moroccan cobra *Naja haje legionis* venom using tandem mass spectrometry. J. Proteom..

[59] Tan K.Y., Tan C.H., Fung S.Y., Tan N.H. (2015). Venomics, lethality and neutralization of *Naja kaouthia* (monocled cobra) venoms from three different geographical regions of Southeast Asia. J Proteom..

[60] Tan N.H., Wong K.Y., Tan C.H. (2017). Venomics of *Naja sputatrix*, the Javan spitting cobra: A short neurotoxin-driven venom needing improved antivenom neutralization. J. Proteom..

[61] Habib A.G., Musa B.M., Iliyasu G., Hamza M., Kuznik A., Chippaux J.-P. (2020). Challenges and prospects of snake antivenom supply in sub-Saharan Africa. PLoS Negl. Trop. Dis..

[62] WHO (2016). WHO Guidelines for the Production, Control and Regulation of Snake Antivenom Immunoglobulins.

[63] Ratanabanangkoon K., Tan K.Y., Pruksaphon K., Klinpayom C., Gutiérrez J.M., Quraishi N.H., Tan C.H. (2020). A pan-specific antiserum produced by a novel immunization strategy shows a high spectrum of neutralization against neurotoxic snake venoms. Sci. Rep..

[64] Ratanabanangkoon K., Tan K.Y., Eursakun S., Tan C.H., Simsiriwong P., Pamornsakda T., Wiriyarat W., Klinpayom C., Tan N.H. (2016). A Simple and Novel Strategy for the Production of a Pan-specific Antiserum against Elapid Snakes of Asia. PLoS Negl. Trop. Dis..

[65] Howard-Jones N. (1985). A CIOMS ethical code for animal experimentation. WHO Chron..

[66] Tan C.H., Wong K.Y., Tan K.Y., Tan N.H. (2017). Venom proteome of the yellow-lipped sea krait, *Laticauda colubrina* from Bali: Insights into subvenomic diversity, venom antigenicity and cross-neutralization by antivenom. J. Proteom..

[67] Finney D.J. (1952). Probit Analysis.

[68] Morais V., Ifran S., Berasain P., Massaldi H. (2010). Antivenoms: Potency or median effective dose, which to use?. J. Venom. Anim. Toxins Incl. Trop. Dis..

[69] Tan K.Y., Tan N.H., Tan C.H. (2018). Venom proteomics and antivenom neutralization for the Chinese eastern Russell’s viper, *Daboia siamensis* from Guangxi and Taiwan. Sci. Rep..

[70] Oh A.M.F., Tan C.H., Ariaranee G.C., Quraishi N., Tan N.H. (2017). Venomics of *Bungarus caeruleus* (Indian krait): Comparable venom profiles, variable immunoreactivities among specimens from Sri Lanka, India and Pakistan. J. Proteom..

[71] Ma J., Chen T., Wu S., Yang C., Bai M., Shu K., Li K., Zhang G., Jin Z., He F. (2019). iProX: An integrated proteome resource. Nucleic Acids Res..

